# Innovative approach for first‐trimester fetal organ volume measurements using a Virtual Reality system: The Generation R *Next* Study

**DOI:** 10.1111/jog.15151

**Published:** 2022-01-29

**Authors:** Clarissa J. Wiertsema, Chalana M. Sol, Annemarie G. M. G. J. Mulders, Eric A. P. Steegers, Liesbeth Duijts, Romy Gaillard, Anton H. J. Koning, Vincent W. V. Jaddoe

**Affiliations:** ^1^ The Generation R Study Group Erasmus University Medical Center Rotterdam The Netherlands; ^2^ Department of Pediatrics Erasmus University Medical Center Rotterdam The Netherlands; ^3^ Departments of Obstetrics and Gynecology Erasmus University Medical Center Rotterdam The Netherlands; ^4^ Department of Pediatrics, Division of Respiratory Medicine and Allergology Erasmus University Medical Center Rotterdam The Netherlands; ^5^ Department of Pediatrics, Division of Neonatology Erasmus University Medical Center Rotterdam The Netherlands; ^6^ Department of Pathology, Clinical Bioinformatics Unit Erasmus University Medical Center Rotterdam The Netherlands

**Keywords:** 3D ultrasound, fetus, first trimester, organ development, reproducibility, virtual reality

## Abstract

**Introduction:**

To investigate the reproducibility of first‐trimester fetal organ volume measurements using three‐dimensional (3D) ultrasound and a Virtual Reality system.

**Methods:**

Within a population‐based prospective cohort study, 3D ultrasound datasets of 25 first‐trimester fetuses were collected by three sonographers. We used the V‐scope application to perform Virtual Reality volume assessments of the fetal heart, lungs, and kidneys. All measurements were performed by two independent researchers.

**Results:**

Intraobserver analyses for volume measurements of the fetal heart, lungs, and kidneys showed intraclass correlation coefficients ≥0.86, mean differences ≤8.3%, and coefficients of variation ≤22.8%. Interobserver analyses showed sufficient agreement for right lung volume measurements, but consistent measurement differences between observers for left lung, heart, and kidney volume measurements (*p*‐values <0.05).

**Conclusion:**

We observed sufficient intraobserver reproducibility, but overall suboptimal interobserver reproducibility for first‐trimester fetal heart, lung, and kidney volume measurements using an innovative Virtual Reality approach. In the current stage, these measurements might be promising for the use in research settings. The reproducibility of the measurements might be further improved by novel post‐processing algorithms.

## Introduction

Early fetal life is a crucial period for organ development.^1^ Previous studies have shown that suboptimal first‐trimester development, measured by crown rump length in women with a known last menstrual period date, is associated with increased risks of adverse fetal and birth outcomes.^2^, ^3^, ^4^, ^5^ First‐trimester fetal growth has also been associated with childhood cardiovascular and respiratory outcomes.^6^, ^7^, ^8^, ^9^ The mechanism underlying these associations might include structural adaptations in early organ development.^10^ Detailed studies on first‐trimester fetal organ development might improve understanding of mechanisms underlying fetal developmental adaptations that may lead to adverse cardiovascular and respiratory outcomes in later life. Thus far, studies on in‐vivo first‐trimester organ development are scare. Three‐dimensional (3D) transvaginal ultrasound allows detailed visualization of first‐trimester anatomy. Virtual Reality approaches enable visualization of 3D ultrasounds as hologram, which offers opportunities for volumetric measurements of complex early pregnancy fetal structures.^11^ Embryonic volume measurements using a region‐growing segmentation algorithm in a Virtual Reality setting have previously shown to be feasible.^11^ These embryonic measurements seem related to fetal growth and birth outcomes.^12^, ^13^ Recently, we have reported that first‐trimester fetal proportion volumetric measurements using a Virtual Reality approach are reproducible.^14^ More detailed measurements of cardiovascular and respiratory tract related organs might be useful in research on fetal developmental adaptations and long‐term cardiovascular and respiratory consequences.^10^

We aimed to develop a novel method for fetal heart, lung, and kidney volume measurements in the late first trimester using a Virtual Reality approach. We assessed the intraobserver and interobserver reproducibility for volume measurements of fetal organs in 25 fetuses in the late first trimester.

## Methods

### Study design

This study was embedded in the Generation R *Next* Study, a population‐based prospective cohort study from preconception onwards in Rotterdam, the Netherlands. Recruitment started in August 2017 and is still ongoing.^14^ Pregnant women were invited to the research center for three appointments in the first trimester of pregnancy, from 7 to 13 weeks of gestation, with an interval of approximately 2 weeks. During these 30‐min visits, 3D ultrasound datasets were obtained to assess embryonic, early fetal and placental development. Around 30 weeks of gestation, participants were invited back to the research centre for a follow‐up ultrasound. All participating women gave written informed consent. The medical ethics committee of the Erasmus University Medical Center approved of this study (MEC‐2016‐589, December 2016). For the current analysis, we focused on 3D ultrasound datasets collected in the late first‐trimester. From March to April 2019, we selected a random group of 25 participants who visited the research center at the Erasmus MC, in whom all the 3D ultrasound data according to the ultrasound study protocol were acquired.

### Gestational age assessment

Gestational age was calculated from the first day of the last menstrual period in spontaneous pregnancies or from oocyte pick‐up plus 14 days in IVF pregnancies. Gestational age was based on crown rump length if the last menstrual period was unknown or gestational age determined by crown rump length differed more than 7 days from the last menstrual period.^14^, ^15^

### Fetal ultrasound examination

All ultrasound scans were performed by three experienced ultra‐sonographers using a Voluson E10 System (GE Healthcare, Zipf, Austria) with a 5–13 MHz transvaginal transducer (RIC6‐12D). The ultrasound settings were predefined to collect high quality ultrasound data in an uniform manner (gain = 0, line filter = low, persistence filter = 2, enhance = 2, dynamic contrast = 6, enhance = 2).^14^ We acquired 3D ultrasound dataset of the fetal trunk under a 40° volume angle while the fetus was not moving. Multiple 3D ultrasound datasets were collected during the examination. To allow proper imaging of the fetal heart and lungs, the 3D ultrasound datasets of the trunk were preferably acquired from the midsagittal plane while the fetus was facing toward the transducer. To allow proper imaging of the fetal kidneys, the 3D ultrasound datasets of the trunk were preferably acquired from the midsagittal plane while the fetus was facing away from the transducer.

### Fetal organ volume measurements

**FIGURE 1:**
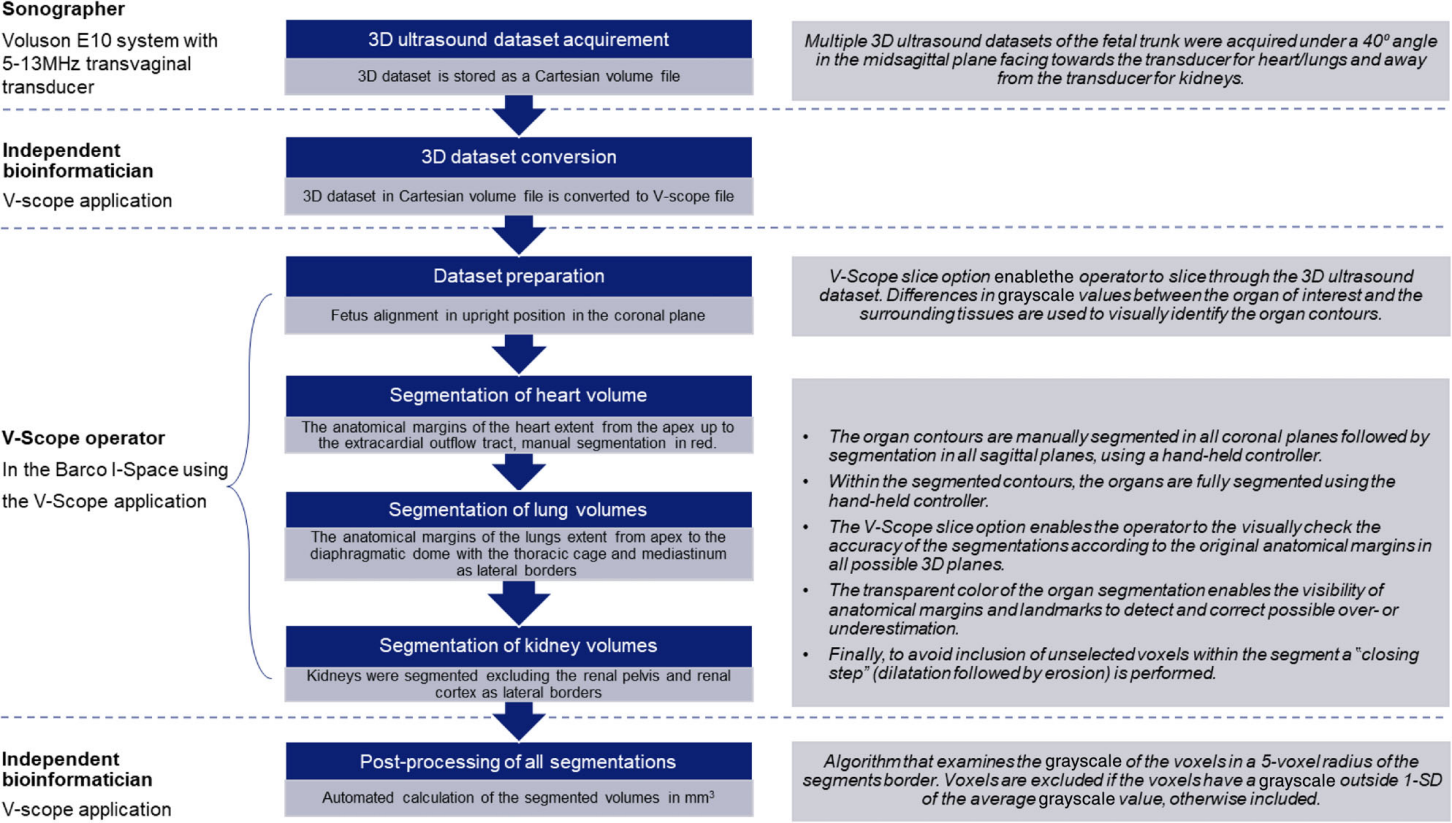
Flowchart of the protocol for segmentation of the organ volumes

**FIGURE 2:**
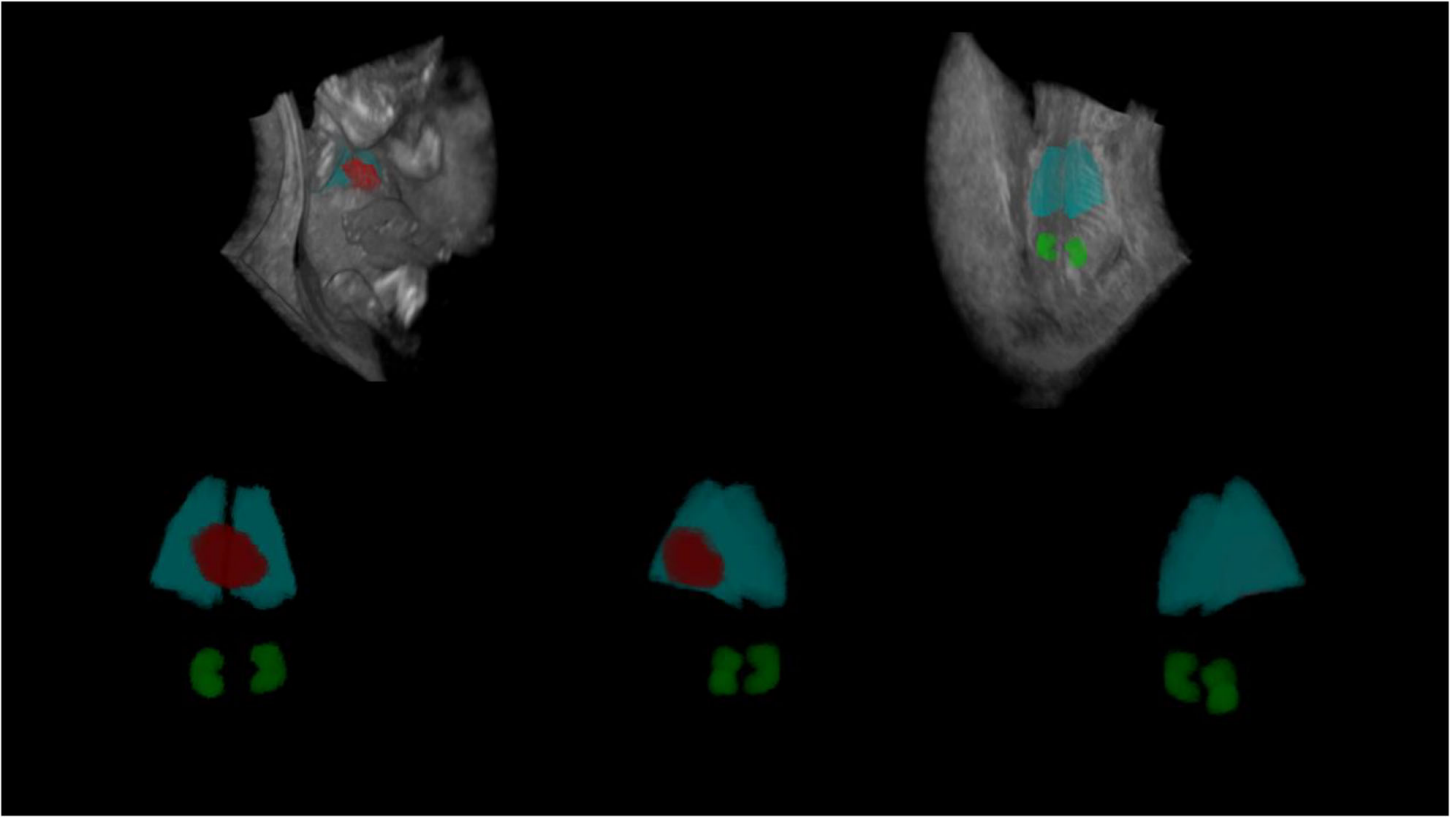
Three‐dimensional view of the segmented heart, lungs, and kidneys of a fetus at 11 weeks and 6 days of gestation in the BARCO I‐Space obtained using the V‐scope application

In the Barco I‐Space, a CAVE™‐like Virtual Reality system, we used the V‐scope volume rendering application for offline analysis of the 3D ultrasound datasets.^16^ The 3D ultrasound datasets were first stored as a Cartesian volume files, and then converted to our own V‐scope file format to allow offline analysis using the V‐Scope application. The V‐Scope application enables accurate volumetric measurements due to detailed depth perception offered by Virtual Reality displays that create a hologram of the 3D ultrasound dataset.^17^, ^18^ With a hand‐held controller, the V‐Scope slice option enables the operator to “slice through” the 3D ultrasound dataset in all preferred 3D planes. Within the current study, differences in grayscale values between the organ of interest and the surrounding tissues were used to visually identify the contours of the fetal heart, lungs, and kidneys. We did not apply a smoothing module to produce a less pixelated image of the 3D ultrasound datasets, as this will reduce the already minimal differences in grayscale value of the various tissues. After identification of the organ contours, the operator manually selects voxels within the 3D ultrasound dataset using a brusher that is adjustable in size. This process results in a “volume segmentation” of the organ of interest. After manual segmentation, all volumes were post‐processed using an algorithm that examines the grayscale of the voxels in a radius of 5 voxels of the segments border: if the voxels have a grayscale outside one standard deviation (SD) of the average grayscale value they were excluded, otherwise they were included. This automated post‐processing algorithm was created to increase the accuracy of the delineation of the anatomical boundaries used for the segmentation. Finally, the organ volume segmentations were automatically calculated in mm^3^ and used for the final analyses. The organ volume measurements were performed using a detailed measurement protocol with instructions on the alignment of the fetus, the plane in which the volume segmentation should be performed, and the size of the brusher. For a detailed description of the measurement procedure, see Figure 1 and Appendix S1 “Protocol for first trimester organ volume measurements using the V‐Scope application.” Figure 2 and Video S1 show a 360° 3D view of the segmented heart, lungs, and kidneys of a fetus at 11 weeks and 6 days of gestation.

The volumetric measurements were performed in a blinded setting by two operators (C.W. and C.S.), who were experienced ultrasonographers with previous experience of performing VR volume measurements using the V‐Scope application. The first operator (C.W.) assessed the overall quality of the datasets, whether movement artifacts or acoustic shadowing were present, and if the region of interest was complete. The dataset of the best quality was used for further offline analyses. Both operators performed the offline measurements twice, with an interval of at least 1 week to prevent recall bias.

### Statistical analysis

We performed the statistical analysis as proposed by Bland and Altman, as we previously described.^14^, ^19^, ^20^ For the intraobserver analysis, the first measurement of each observer was compared with their second measurement. For the interobserver analysis, the mean of the two measurements of the first observer was compared to the mean of the two measurements of the second observer using similar calculations. First, we plotted the measurements with the line of equality to give an initial sense of the degree of agreement.^19^ Second, intraclass correlation coefficients (ICC) with a 95% confidence interval and the coefficients of variation (CV) were calculated for each measurement to evaluate consensus within each observer and between observers.^19^ Third, intraobserver and interobserver variability was quantified calculating the mean difference in percentage measurement error with the 95% limits of agreement (mean difference (%) ±1.96 SD) for all the fetal organ volume measurements. Within the limits of agreement, the measurements within and between observers can be assumed to be interchangeable.^15^ A paired sample *t*‐test was performed to identify possible consistent intra‐ or interobserver differences. Lastly, we plotted the mean differences in percentage measurement error with the 95% limits of agreement. These so‐called Bland and Altman plots were specifically provided to visualize that the agreement for the volumetric measurements does not depend on organ size. We consider the ICC, CV, mean difference, and the limits of agreement as our main outcomes of interest. We decided a priori that an ICC >90%, a CV <10%, a mean difference <10%, and limits of agreement within ±10% were considered to be proof of excellent agreement.^21^ Importantly, an acceptable mean difference and acceptable limits of agreement are not a statistical but a more subjective consideration.^15^ To establish that the measurements are useable for future association studies, we considered that the limits of agreement should deviate a maximum of 10% from the mean difference, which indicates that 95% of all differences should be within the ±10% measurement error range.^20^ Statistical analyses were performed using IBM SPSS, version 25.

## Results

**FIGURE 3:**
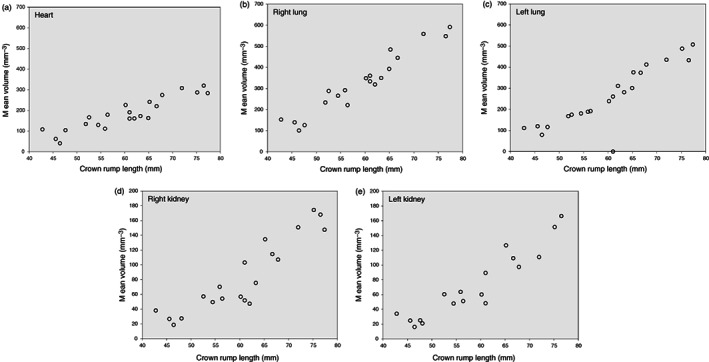
General characteristics of the study populations: Volume measurements of (a) heart, (b) right lung, (c) left lung, (d) right kidney, and (e) left kidney of the study subjects in relation to crown rump length

Characteristics of the participants are shown in Table 1. The gestational age ranges between 10.7 and 13.3 weeks. Descriptives of fetal organ volume measurements are shown in Table 2. In these 25 participants, a total of 84 3D ultrasound datasets of the fetal trunk were available, on average 3.4 per participant. The number of datasets in which both observers were able to perform the fetal organ volume measurements was 22 out of 25 (88%) for heart and right lung, 21 out of 25 (84%) for left lung, and 20 and 18 out of 25 (72–80%) for kidney volumes. Figure 3 shows that the average fetal organ volumes increase with fetal crown rump length.

**TABLE 1:** Participant characteristics (*n* = 25)

	Median (IQR)/*n* (%)
Maternal age (years)	32.0 (31, 36)
Maternal Body mass index (kg/m^2^)	22.7 (20.8, 26.3)
Gestational age (weeks)	12.1 (11.4, 12.7)
Crown Rump Length (mm)	60.2 (50.0, 65.9)
Conception mode	
Spontaneously conceived (%)	23 (92)
In vitro fertilization (%)	1 (4)
Ovulation induction (%)	1 (4)

**TABLE 2:** Descriptives of fetal organ volume measurements for both observers (*n* = 25)

Volumetric measurement	Observer	Measurement	Mean (minimum, maximum) (mm^3^)
Heart	1	1	206 (39, 409)
		2	217 (58, 370)
	2	1	156 (29, 305)
		2	158 (39, 317)
Right lung	1	1	373 (92, 811)
		2	372 (119, 735)
	2	1	361 (103, 90)
		2	354 (90, 672)
Left lung	1	1	269 (74, 543)
		2	260 (67, 514)
	2	1	288 (99, 505)
		2	279 (78, 512)
Right kidney	1	1	102 (28, 245)
		2	102 (31, 227)
	2	1	65 (6, 138)
		2	65 (10, 120)
Left kidney	1	1	89 (20, 216)
		2	90 (21, 214)
	2	1	54 (10, 115)
		2	57 (13, 121)

### Reproducibility analyses

**FIGURE 4:**
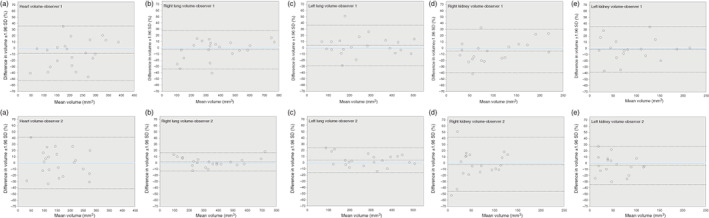
Bland and Altman plots of intraobserver agreement with corresponding limits of agreement in percentage of the mean ± 1.96 SD for (1) Observer 1 and (2) Observer 2 for volume measurements of: (a) heart, (b) right lung, (c) left lung, (d) right kidney, and (e) left kidney

For the intraobserver analyses, the heart, lung, and kidney volumes measurements lie close to and evenly scattered around the line of equality for both observers, suggesting acceptable intraobserver differences (Figures S1 and S2). Table 3 shows the ICC, CV, and mean difference with corresponding limits of agreement for the intraobserver analyses. For volumetric measurements of the heart, intraobserver ICCs were 0.86 and 0.90, CVs were 22.8% and 21.8% for Observers 1 and 2, respectively. Intraobserver ICCs for all other measurement were above 0.95 and CVs below 19.6%. The *p*‐values obtained from the paired sample *t*‐tests for all measurements of both observers were >0.05, suggesting no consistent intraobserver measurement differences. Figure 4 shows that the agreement does not depend on organ size for all volumetric measurements for both observers. The mean differences for all measurements were below 8.3%. Limits of agreement ranged from −52.3% to 41.9%.

**TABLE 3:** Intraobserver agreement of fetal organ volume measurements for both observers (*n* = 25)

Volumetric measurement	Observer	ICC (95% CI)	CV (%)	Mean difference (LLOA, ULOA) (%)	Paired sample *t*‐test intra‐observer (*p*‐value)
Heart	1	0.86 (0.71, 0.94)	22.8	−8.3 (−52.3, 35.8)	0.29
	2	0.90 (0.77, 0.96)	21.8	−0.1 (−41.6, 41.5)	0.77
Right lung	1	0.96 (0.91, 0.98)	14.4	−3.0 (−34.3, 28.4)	0.94
	2	0.98 (0.96, 0.99)	9.38	1.7 (−12.9, 16.3)	0.36
Left lung	1	0.96 (0.90, 0.98)	15.0	3.9 (−28.7, 36.4)	0.28
	2	0.98 (0.95, 0.99)	9.16	4.3 (−15.6, 24.3)	0.13
Right kidney	1	0.95 (0.88, 0.98)	19.6	−4.8 (−39.8, 30.3)	0.96
	2	0.97 (0.93, 0.99)	14.9	−2.0 (−46.0, 41.9)	0.90
Left kidney	1	0.96 (0.90, 0.99)	18.0	−1.6 (−38.3, 35.2)	0.83
	2	0.97 (0.92, 0.99)	14.8	−3.4 (−34.2, 27.3)	0.17

Abbreviations: CI, confidence interval; CV, coefficient of variation; ICC, intraclass correlation coefficient; LLOA, lower limit of agreement; ULOA, upper limit of agreement.

**FIGURE 5:**
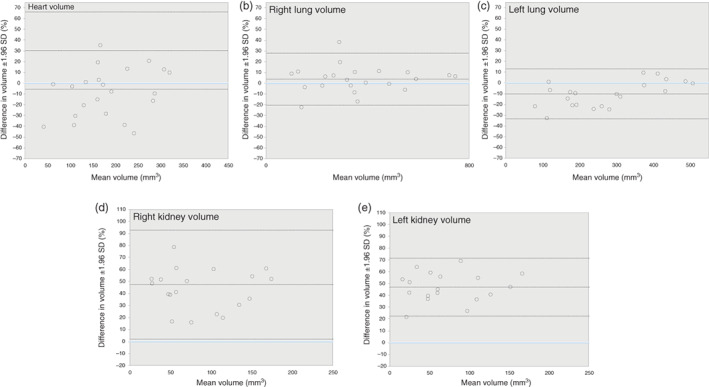
Bland and Altman plots of interobserver agreement with corresponding limits of agreement in percentage of the mean ± 1.96 SD for volume measurements of: (a)heart, (b) right lung, (c) left lung, (d) right kidney, and (e) left kidney

For the interobserver analyses, the measurements of the lung volumes lie in close proximity to the line of equality, suggesting acceptable interobserver differences for these volumetric measurements (Figure S3). The measurements of the heart and kidney volumes all lie below the line of equality, suggesting structural interobserver differences for these volumetric measurements (Figure S3). Table 4 shows the ICC, CV, and mean difference with corresponding limits of agreement for the interobserver analyses. Interobserver ICCs were 0.98 and 0.97, CVs were 10.6 and 10.3 for volumetric measurements of the right and left lung volumes, respectively. Interobserver ICCS for heart, left kidney, and right kidney volumes ranged from 0.68 to 0.71. The *p*‐values obtained from the paired sample *t*‐tests for heart, left lung, right and left kidney were <0.05, indicating consistent interobserver measurement differences for these volumetric measurements. Figure 5 shows that the mean differences for the right and left lung volumes were below 10.0%, with limits of agreement ranging from −33.3% to 28.0%. The mean differences for heart, right and left kidney volume ranged from 30.3 to 47.4, with limits of agreement ranging from −33.3% to 71.6%.

**TABLE 4:** Interobserver agreement of fetal organ volume measurements (*n* = 25)

Volumetric measurement	*n* (%)^a^	ICC (95% CI)	CV (%)	Mean difference (LLOA, ULOA) (%)	Paired sample *t*‐test (*p*‐value)
Heart	22 (88)	0.71 (−0.03, 0.91)	22.7	30.3 (−5.6, 66.2)	<0.01
Right lung	22 (88)	0.98 (0.94, 0.99)	10.6	3.8 (−20.4, 28.0)	0.08
Left lung	21 (84)	0.97 (0.88, 0.99)	10.3	−10.0 (−33.3, 12.9)	0.01
Right kidney	20 (80)	0.69 (−0.06, 0.90)	32.8	47.4 (2.0, 92.7)	<0.01
Left kidney	18 (72)	0.68 (−0.06, 0.91)	34.5	47.0 (22.5, 71.6)	<0.01

Abbreviations: CI, confidence interval; CV, coefficient of variation; ICC, intraclass correlation coefficient; LLOA, lower limit of agreement; ULOA, upper limit of agreement.

^a^ Number and percentage of datasets in which both observers could perform the measurements.

## Discussion

### Main findings

We evaluated a novel Virtual Reality approach to measure first‐trimester fetal heart, lung, and kidney volumes using 3D ultrasound datasets that were acquired in the late first trimester. We observed sufficient intraobserver reproducibility for volumetric measurements of the fetal heart, lungs, and kidneys. We also observed sufficient interobserver reproducibility for volume measurements of the fetal right lung, but suboptimal interobserver reproducibility for volume measurements of the fetal heart, left lung, and both kidneys.

### Interpretation of main findings

Quantitative estimation of first‐trimester development is currently performed by traditional ultrasound length measures, most importantly using the crown rump length.^15^ Volumetric measurements might be more sensitive parameters for first‐trimester growth and development, since the increase in first‐trimester fetal volume is cubic with a linear increase in fetal length.^11^ Over recent years, volume measurement techniques using 3D ultrasound datasets in combination with Virtual Reality have been introduced and seem to be feasible for several first‐trimester volume measurements.^12^ This Virtual Reality technique offers depth perception in high‐resolution and therefore improves the visualization of complex anatomical structures, when compared to regular two‐dimensional displays.^17^

We have recently reported that first‐trimester fetal proportion volumetric measurements are feasible and reproducible.^14^ More detailed measurements on fetal organ systems might be useful for research in the field of Developmental Origins of Health and Disease, as early fetal developmental adaptations in response to an adverse intrauterine environment might directly affect long term health and disease.^8^ We developed a novel approach for volume measurements of the first‐trimester fetal heart, lungs, and kidneys using 3D ultrasound datasets of the fetal trunk acquired in the late first trimester of pregnancy. Our Virtual Reality approach shows promising results for one observer as indicated by high ICCs, CVs around 10% and mean differences <10% for all measurements, although the limits of agreement remain relatively broad. Despite good intraobserver agreement, our method provided inconsistent results in a setting with two observers. Interobserver analyses showed sufficient agreement for fetal right lung volume measurements but structural measurement differences for the heart, left lung, and both kidney volume measurements. The interobserver agreement for the left lung volumetric measurement appeared sufficient as indicated by an ICC of 0.97, and CV and mean difference around 10%, but as the interobserver differences seem consistent we do not consider the agreement for the volume measurements of the left lung as sufficient. The percentage of cases in which both observers could perform the measurements ranged from 72% to 88%, which can be considered a relatively low success percentage. We consider this success percentage sufficiently high for research purposes in large observational cohort studies, but currently insufficient for the use of these measurements in clinical practice.

The suboptimal interobserver reproducibility, most prominently for kidney volumes, can be explained by several factors. First, the measured absolute volumes are extremely small and therefore only allow for minor measurement differences. Second, a large part of this measurement technique involves manual segmentation as it is not possible to automate the recognition of these small but complex anatomical structures. Unfortunately, ultrasound data tend to be noisy, which results in a lack of clear demarcation of the fetal organs, and therefore results in larger interobserver differences. For accurate segmentation of the lungs, the whole diaphragm, the thoracic cage, and the heart needs to be clearly visible. This may explain the sufficient interobserver agreement for the fetal right lung, with a relatively large volume compared to the other organs and a clear demarcation at the borders with the diaphragm and ribcage. Although the kidneys are easily detectible in the late first trimester, the demarcation from the surrounding intestines and liver remains difficult to distinguish in our ultrasound data. Similar difficulties are present regarding segmentation of the fetal heart during the late first trimester.^22^ These difficulties highlight the importance for acquisition of high‐quality 3D ultrasound datasets. For optimal 3D ultrasound acquisition, we used a state‐of‐the‐art ultrasound machine with a high‐frequent transvaginal transducer. The acquisition was performed when the anatomical structures of interest were clearly visible without fetal movements. To our knowledge, only one previous study group developed a method to measure volumes of fetal heart and lungs in fetuses at 12 to 32 weeks gestation using the VOCAL method.^23^ Although the researchers indicate that the reproducibility for these measurement at 12 to 13 weeks gestation was sufficient, they only provide absolute measurement differences and limits of agreement in mL, and do not provide ICC and CV values. This makes it hard to establish if these measurement with VOCAL were truly reproducible in the first trimester, and makes comparison to our study results difficult.^23^ We are not aware of any other studies focused on measurements of the kidney volume in the first trimester.

In comparison to previous studies using a Virtual Reality approach for volumetric measurements in early pregnancy, we found lower interobserver reproducibility.^11^, ^14^, ^18^, ^24^ This is most likely due to a larger role for automated segmentation in these previous studies, compared to the current study. To perform the measurements described in this study, differences in grayscale values between the organ and the surrounding tissues were used to identify the organs, followed by manual segmentation of the organ volume. The operators in this study were experienced sonographers that had previous experience with the V‐scope application, but they still considered this method difficult and time‐consuming. The manual segmentation was followed by an automated post‐processing method that takes into account grayscale differences of voxels at the borders of the segmentation. In the future, we hope to further improve automated post‐processing steps to improve the utility of this method, and reduce intra‐ and interobserver measurement differences. Our primary step is the implementation of adaptive active contour tracking using a snake algorithm in the V‐scope application.^25^ The snake algorithm starts with a rough manual delineation of the object to be segmented and uses an automatic energy minimizing approach where this segmentation is pulled toward object contours, but at the same time resists deformation. This method is already used for image analysis purposes in other biomedical fields and shows high segmentation accuracy.^26^, ^27^, ^28^, ^29^

The method presented in this paper does not seem suitable for clinical purposes in the current stage but might be adequate for the use in large‐scale population‐based research settings such as the Generation R *Next* Study. Within this study, it is not specifically our aim to provide normative values for fetal organ growth but we aim to provide insights in the influence of periconceptional factors on early organ development. If the organ volume measurements are conducted by a small group of operators, operator adjusted *Z* scores of organ volumes can be calculated to reduce the problems with interobserver differences within the statistical analyses. As the potential measurement error would be non‐differential, the use of these operator adjusted *Z* scores would not likely lead to biased estimates in association studies. Within the Generation R *Next* Study, we will assess whether early fetal organ size is associated with parental lifestyle and health during the preconception phase.^14^ With the aim to do a long‐term follow‐up of the children that participate in the Generation R *Next* Study, these novel measurements might also be useful to investigate whether early fetal alterations in organ size influence cardiovascular and respiratory health during childhood.

In conclusion, we observed sufficient intraobserver reproducibility for volume measurements of the fetal heart, lungs, and kidneys using a novel Virtual Reality approach. The interobserver reproducibility seems suboptimal. In the current stage, these measurements might be promising for the use in research settings, but not for clinical purposes. The reproducibility of the measurements might be further improved by novel post‐processing algorithms.

## Author Contributions

Clarissa J. Wiertsema, Chalana M. Sol, Anton H. J. Koning, Annemarie G. M. G. J. Mulders, Romy Gaillard, and Vincent W. V. Jaddoe were responsible for design and planning of the study. Clarissa J. Wiertsema, Chalana M. Sol, and Anton H. J. Koning were responsible for the development of the fetal organ volume measurements. Clarissa J. Wiertsema and Chalana M. Sol were responsible for the data collection. Clarissa J. Wiertsema, Chalana M. Sol, Romy Gaillard, and Vincent W. V. Jaddoe had full access to all of the data and take responsibility for the integrity of the data and the accuracy of the data analysis. Clarissa J. Wiertsema wrote the main manuscript text. Chalana M. Sol, Annemarie G. M. G. J. Mulders, Eric A. P. Steegers, Liesbeth Duijts, Anton H. J. Koning, Romy Gaillard, and Vincent W. V. Jaddoe were responsible for critical review of the manuscript. All authors approved the final manuscript and agree to be accountable for all aspects of the work.

## Disclosure

No relevant financial, personal, political, intellectual, or religious conflicts of interest are declared.

## Supporting information


**Video S1** 360° three‐dimensional view of the segmented heart, lungs, and kidneys of a fetus at 11 week and 6 days of gestation in the BARCO I‐Space obtained using the V‐scope application.

**Appendix** **S1**. Protocol for first trimester fetal organ volume measurements using the V‐Scope application.

**Appendix** **S** **2**. Supplementary figures.

## Data Availability

The data that support the finding of this study are available from the corresponding author upon reasonable request.
