# Supplementary material for: Innovative approach for first‐trimester fetal organ volume measurements using a Virtual Reality system: The Generation R Next Study
Source: J Obstet Gynaecol Res. 2022 Jan 29;48(3):599–609. doi: 10.1111/jog.15151 (PMC9306822; doi:10.1111/jog.15151)
Supplement: Supplementary file 2 — Appendix S1. Protocol for first trimester fetal organ volume measurements using the V‐Scope application. [file JOG-48-599-s002.pdf]

**Supplemental Material**

# Innovative approach for first-trimester fetal organ volume measurements using a Virtual Reality system: The Generation R *Next* Study

Clarissa J. Wiertsema[a,b], Chalana M. Sol[a,b], Annemarie G.M.G.J. Mulders[c], Eric A.P. Steegers[c], Liesbeth Duijts[d,e], Romy Gaillard MD PhD[a,b], Anton H. J. Koning[a,f], Vincent W.V. Jaddoe MD PhD[a,b]

[a] The Generation R Study Group, Erasmus University Medical Center, Rotterdam, The Netherlands.
[b] Department of Pediatrics, Erasmus University Medical Center, Rotterdam, The Netherlands.
[c] Departments of Obstetrics and Gynecology, Erasmus University Medical Center, Rotterdam, The Netherlands.
[d] Department of Pediatrics, Division of Respiratory Medicine and Allergology, Erasmus University Medical Center, Rotterdam, The Netherlands.
[e] Department of Pediatrics, Division of Neonatology, Erasmus University Medical Center, Rotterdam, The Netherlands.
[f] Department of Pathology, Clinical Bioinformatics Unit, Erasmus University Medical Center, Rotterdam, The Netherlands.

**Short title**: Fetal organ measurement using VR

**Corresponding author:** Vincent W.V Jaddoe, The Generation R Study Group (Na 29-15), Erasmus Medical Center, P.O. Box 2040, 3000 CA Rotterdam, the Netherlands; phone: +31 (0)10 704 3405; fax: +31 (0)10 704 4619; e-mail: v.jaddoe@erasmusmc.nl.

| **Page** | |
|---|---|
| 1 | Protocol for first trimester fetal organ volume measurements using the V-Scope application. |

**Protocol for first trimester fetal organ volume measurements using the V-Scope application.**

The three-dimensional dataset containing the trunk of the fetus is visualized in the BARCO I-Space using the V-scope application.[1, 2] The first observer (C.W.) assesses the overall quality of the 3D ultrasound datasets, whether movement artefacts or acoustic shadowing are present, and if the region of interest is complete. The dataset of the best quality is used for further offline analyses. The volume measurements of the heart and lungs should be performed within the same dataset. The volume measurements of the kidneys can be performed within a different 3D ultrasound dataset, if visualization of the kidneys is better within this dataset. The fetus is aligned in an upright position facing towards the operator (coronal plane) before every measurement. The volumetric measurements are executed in a consecutive order: 1) heart, 2) right lung, 3) left lung, 4) right kidney, and 5) left kidney. Measurement could only be performed if the required anatomical margins can be visualized. Voxels can be manually selected and deleted using a brusher that is adjustable in size. A small brush radius is used for all segmentations to allow detailed tracing of the organ contour. The V-Scope slice option enables the observer to check if the voxels are selected according to the original anatomical margins. The transparent color of the segmentation enables the visibility of anatomical landmarks during the segmentation process. This enables detection of segmentation errors that would indicate under- or overestimation of the volumetric measurement. If errors are detected by the operator, corrections can be made accordingly. All volume measurements are post-processed to increase the accuracy of the delineation of the anatomical boundaries used for the segmentations.

## 1. Volume of the fetal heart

**Measurement**: Volume of the fetal heart.

**Segmentation colour**: Red (opaque/transparent).

**Anatomical margins**: Cardial apex up to the extracardial outflow tract.

**Conditions**: If the anatomical margins of the fetal heart are not clearly visible due to extensive acoustic shadowing or low-quality data, the volume measurement cannot be performed.

**Process specification**:

1. **Manual segmentation**:

- The ‘red opaque’ segmentation colour is selected.
- The contour of the heart was manually drawn in the coronal planes, using a brush radius size 3.
- The contour of the heart was manually drawn in the sagittal planes, using a brush radius size 3.
- The heart volume is filled within the earlier drawn margins.
- During the above described process the V-scope ‘zoom option’ should be used for detailed visualisation of anatomical margins.
- The ‘red transparent’ segmentation colour is selected to enables the visibility of anatomical landmarks during the following steps.
- The V-Scope ‘slice option’ is used to check if the voxels are selected according to the original anatomical margins.
- Segmentation corrections can be made from different planes using the spherical brusher in combination with the ‘deselect voxels’ or ‘select voxels’ options in V-scope; or the segment can be reloaded and the protocol step can be repeated.

2. **Automatic segmentation**: The V-scope ‘dilate option’ and ‘erode option’ are used consecutively, to avoid inclusion of unselected voxels within the segment. Only after this automated processing step, the right lung volume can be segmented.
3. The obtained segmentation is saved.

**2.Volume of the fetal lungs**

**Measurements**: Volumes of the right and left fetal lungs.

**Segmentation colours**: Cyan (opaque/transparent) for right lung, yellow (opaque/transparent) for left lung.

**Anatomical margins**: Apex to the diaphragmatic dome, with the thoracic cage and mediastinum as lateral borders.

**Conditions**: If the anatomical margins of the fetal lungs are not clearly visible due to extensive acoustic shadowing or low-quality data, the volume measurement cannot be performed.

**Process specification**:

1. **Manual segmentation of the right lung**:
    - The ‘cyan opaque’ segmentation colour is selected.
    - The contour of the right lung was manually drawn in the coronal planes, using a brush radius size 3.
    - The contour of the right was manually drawn in the sagittal planes, using a brush radius size 3.
    - The heart volume is filled within the earlier drawn margins.
    - During the above described process the V-scope ‘zoom option’ should be used for detailed visualisation of anatomical margins.
    - The ‘cyan transparant’ segmentation colour is selected to enables the visibility of anatomical margins during the following steps.
    - The V-Scope ‘slice option’ is used to check if the voxels are selected according to the original anatomical margins.
    - Segmentation corrections can be made from different planes using the spherical brusher in combination with the ‘deselect voxels’ or ‘select voxels’ options in V-scope; or the segment can be reloaded and the protocol step can be repeated.
2. **Automatic segmentation of the right lung**: The V-scope ‘dilate option’ and ‘erode option’ are used consecutively, to avoid inclusion of unselected voxels within the segment. Only after this automated processing step, the right lung volume can be segmented.
3. The obtained segmentation is saved.
4. The above described process is followed for the segmentation of the left lung using the ‘yellow opaque’ and the ‘yellow transparent’ segmentation colours.

**<u>3. Volume of the fetal kidneys</u>**

**Measurements**: Volumes of the right and left fetal kidneys.

**Segmentation colours**: Magenta transparent for right kidney, green transparent for left kidney.

**Anatomical margins**: The margins of the renal capsule excluding the renal pelvis.

**Conditions**: If the anatomical margins of the fetal kidneys are not clearly visible due to extensive acoustic shadowing or low-quality data, the volume measurement cannot be performed.

**Process specification**:

1. **Manual segmentation**:
    1. The ‘magenta transparent’ segmentation colour is selected to enables the visibility of anatomical landmarks during the following steps.
    2. The contour of the right kidney was manually drawn in the coronal planes, using a brush radius size 3.
    3. The contour of the right kidney was manually drawn in the sagittal planes, using a brush radius size 3.
    4. The right kidney volume is filled within the earlier drawn margins.
    5. During the above described process the V-scope ‘zoom option’ should be used for detailed visualisation of anatomical margins.
    6. The V-Scope ‘slice option’ is used to check if the voxels are selected according to the original anatomical margins.
    7. Segmentation corrections can be made from different planes using the spherical brusher in combination with the ‘deselect voxels’ or ‘select voxels’ options in V-scope; or the segment can be reloaded and the protocol step can be repeated.
2. **Automatic segmentation**: The V-scope ‘dilate option’ and ‘erode option’ are used consecutively, to avoid inclusion of unselected voxels within the segment. Only after this automated processing step, the right lung volume can be segmented.
3. The obtained segmentation is saved.

4. The above described process is followed for the segmentation of the left kidney using the ‘green transparent’ segmentation colour.

**Step 4. Post-processing procedure**

All segmentations are then post-processed using an optimization algorithm that examines the greyscale of the voxels in a radius of 5 voxels of the segments border: if the voxels have a greyscale outside one standard deviation (SD) of the average greyscale value they were excluded, otherwise they are included. The SD of the 26-connected neighborhood of the included border-voxels has to be equal or smaller than the average SD of all the voxels within the segment. This automated post-processing step increases the accuracy of the delineation of the anatomical boundaries used for the segmentation. The segmented volumes were automatically calculated in $mm^3$ and used as final organ volumes.

**References**

1. Cruz-Neira. Surround-screen projection-based virtual reality: the design and implementation ofthe CAVE (tm). *Proceedings of the 20th annual conference on computer graphics and interactive techniques*. 1993, p. 135-42
2. Koning AH, Rousian M, Verwoerd-Dikkeboom CM, Goedknegt L, Steegers EA and van der Spek PJ. V-scope: design and implementation of an immersive and desktop virtual reality volume visualization system. *Stud Health Technol Inform*. 2009; 142: 136-8.
