# Supplementary material for: Innovative approach for first‐trimester fetal organ volume measurements using a Virtual Reality system: The Generation R Next Study
Source: J Obstet Gynaecol Res. 2022 Jan 29;48(3):599–609. doi: 10.1111/jog.15151 (PMC9306822; doi:10.1111/jog.15151)

Supplemental Figures

Innovative approach for first-trimester fetal organ volume measurements using a Virtual Reality system: The Generation R *Next* Study

Clarissa J. Wiertsema[a,b], Chalana M. Sol[a,b], Annemarie G.M.G.J. Mulders[c], Eric A.P. Steegers[c], Liesbeth Duijts[d,e], Romy Gaillard MD PhD[a,b], Anton H. J. Koning[a,f], Vincent W.V. Jaddoe MD PhD[a,b]

[a] The Generation R Study Group, Erasmus University Medical Center, Rotterdam, The Netherlands.
[b] Department of Pediatrics, Erasmus University Medical Center, Rotterdam, The Netherlands.
[c] Departments of Obstetrics and Gynecology, Erasmus University Medical Center, Rotterdam, The Netherlands.
[d] Department of Pediatrics, Division of Respiratory Medicine and Allergology, Erasmus University Medical Center, Rotterdam, The Netherlands.
[e] Department of Pediatrics, Division of Neonatology, Erasmus University Medical Center, Rotterdam, The Netherlands.
[f] Department of Pathology, Clinical Bioinformatics Unit, Erasmus University Medical Center, Rotterdam, The Netherlands.

Short title: Fetal organ measurement using VR

Corresponding author: Vincent W.V Jaddoe, The Generation R Study Group (Na 29-15), Erasmus Medical Center, P.O. Box 2040, 3000 CA Rotterdam, the Netherlands; phone: +31 (0)10 704 3405; fax: +31 (0)10 704 4619; e-mail: v.jaddoe@erasmusmc.nl.

**Page**		
1	Figure S1	Measurements of observer 1 plotted with line of equality for all fetal organ volume measurements
2	Figure S2	Measurements of observer 2 plotted with line of equality for all fetal organ volume measurements
3	Figure S3	Measurements of observer 1 plotted against measurements of observer 2 with line of equality for all fetal organ volume measurements

Figure S1. Measurements of the observer 1 plotted with line of equality for volume measurement of: A) Heart, B) Right lung, C) Left lung, D) Right kidney, E) Left kidney.

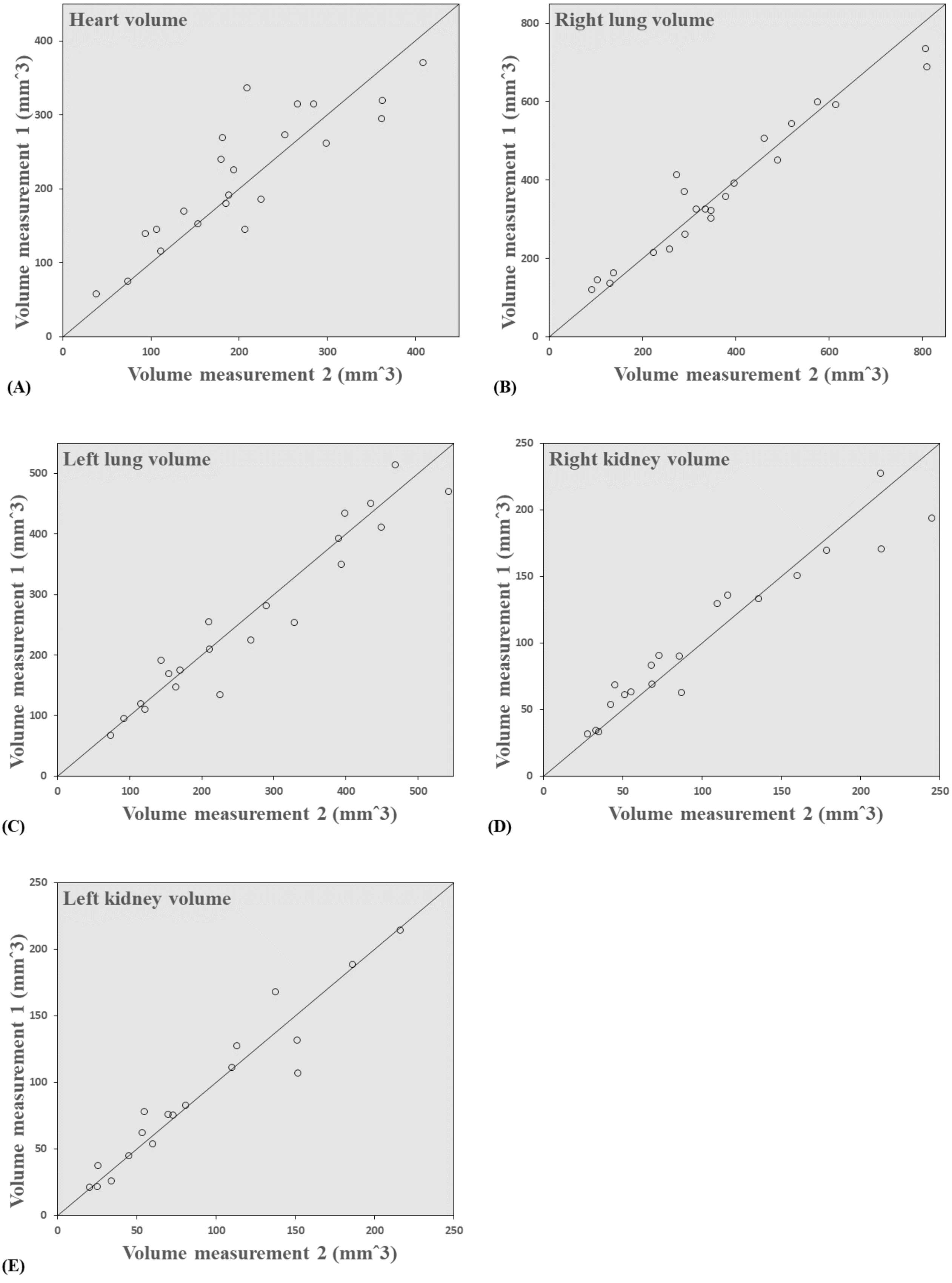

Figure S2. Measurements of observer 2 plotted with line of equality for volume measurement of: A)Heart, B) Right lung, C) Left lung, D) Right kidney, E) Left kidney.

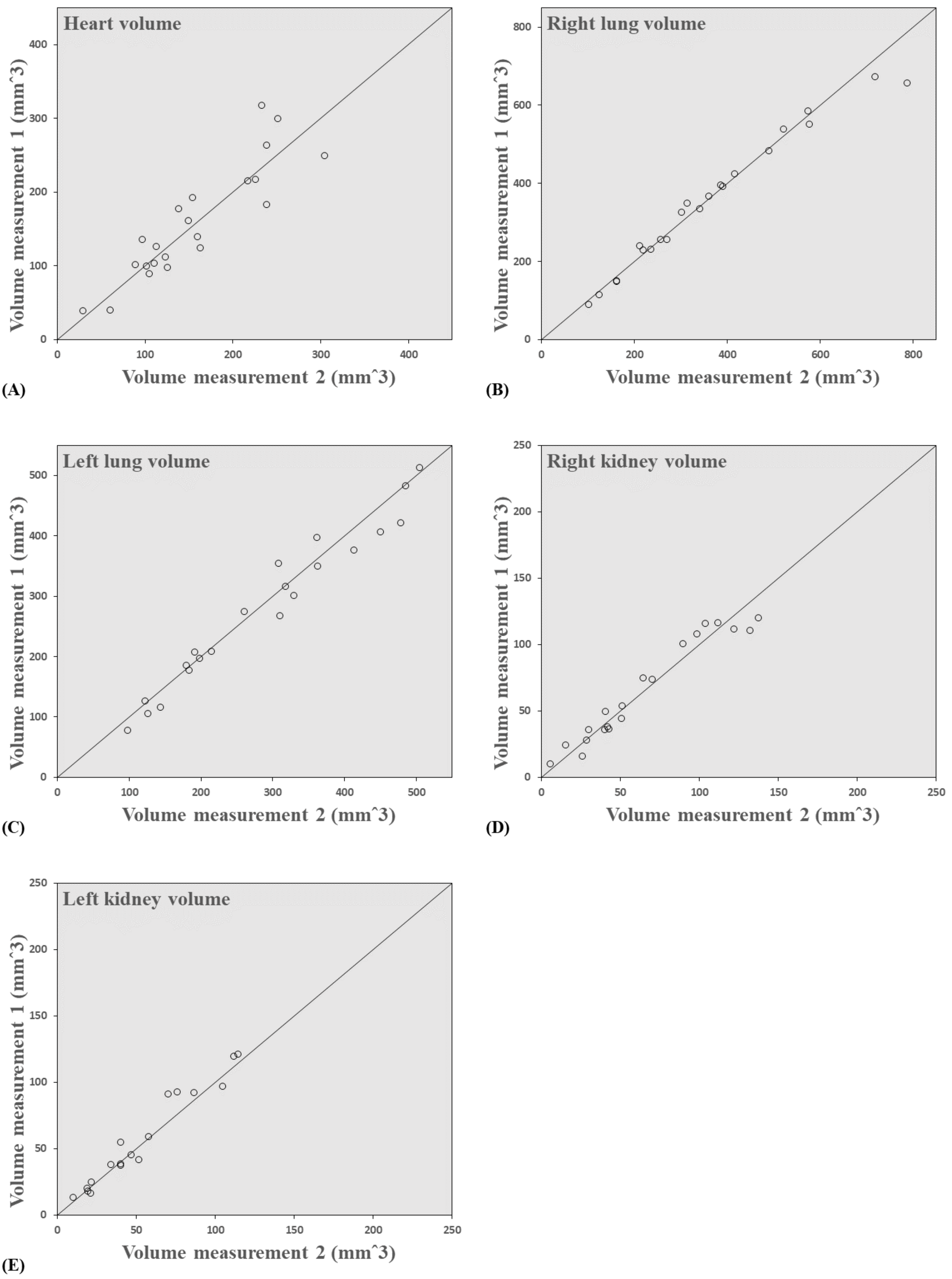

Figure S3. Measurements of observer 1 plotted against measurements of observer 2 with line of equality for all fetal organ volume measurements.

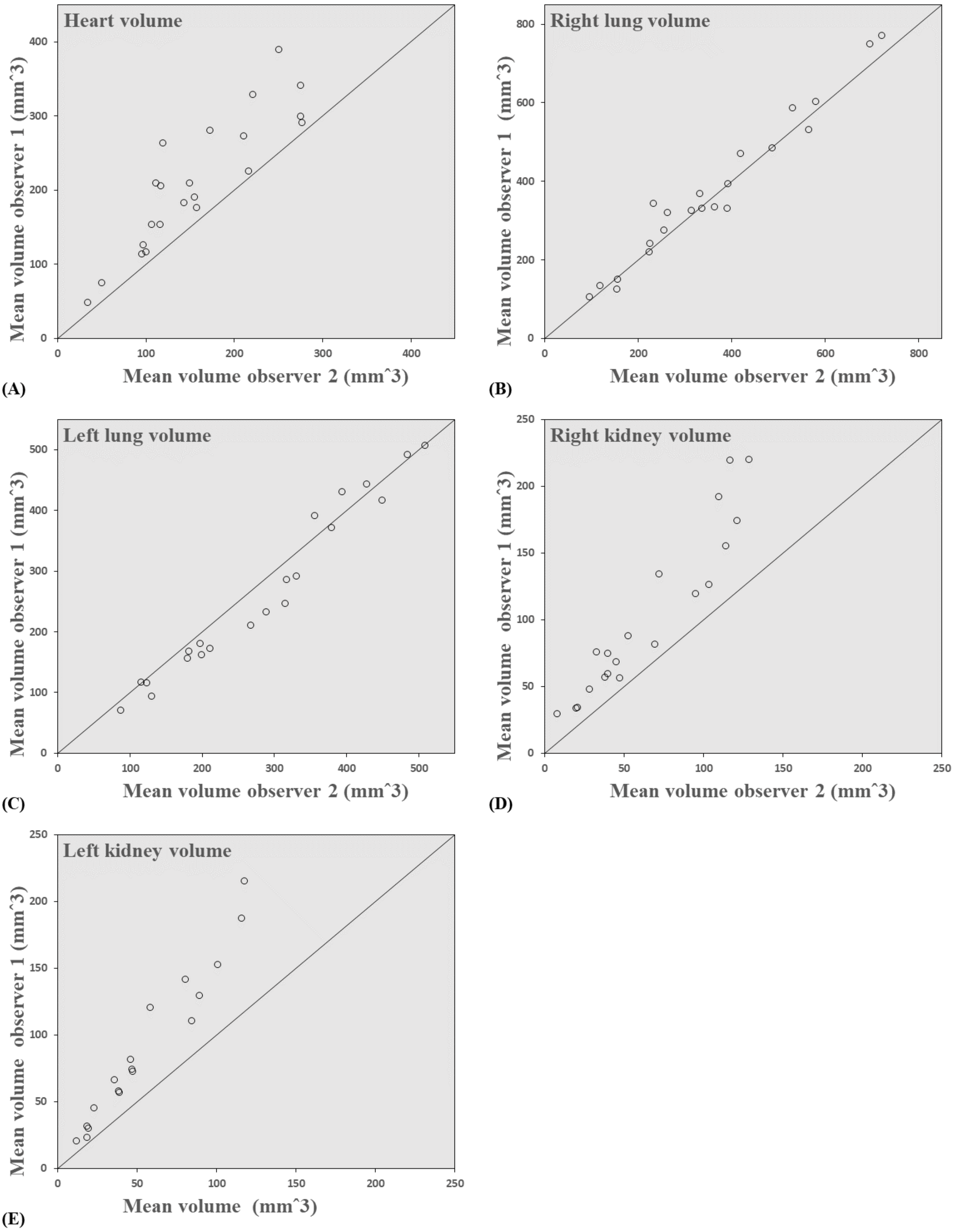

Supplement: Supplementary file 3 — Appendix S 2. Supplementary figures. [file JOG-48-599-s003.pdf]
